# Supplementary material for: Are carbon emissions trading and green financial instruments synergistic? -Comprehensive quantitative research based on content analysis
Source: PLoS One. 2024 Mar 7;19(3):e0298601. doi: 10.1371/journal.pone.0298601 (PMC10919601; doi:10.1371/journal.pone.0298601)
Supplement: S1 Data — (ZIP) [file pone.0298601.s001.zip › Data/地级市Z_F工作报告.zip!/║╙─╧/╫ñ┬φ╡Ω╩╨/└┤╫╘╣1⁄2╓┌║┼í╛┬φ┐╦╩2╛▌═°í┐_2003.docx]

各位代表:

现在，我代表市人民政府，向大会作政府工作报告，请予审议，并请各位政协委员提出意见。

折叠编辑本段2002年工作完成情况

2002年，在省委、省政府和市委的领导下，在市人大、市政协的监督和支持下，市政府带领全市人民,团结奋进，开拓创新，扎实工作，较好地完成了市一届人大二次会议确定的各项任务。

(一)国民经济保持快速增长。预计全年完成国内生产总值332亿元，比上年增长10.2%，好于年初的预期目标。农村经济全面发展，粮食总产再创历史新高，达494万吨，比上年增长19.4%;畜牧、林果、蔬菜、水产业稳步发展;乡镇企业产值利税保持较快增长。工业速度效益稳步提高，全年完成工业增加值113亿元，比上年增长9.6%，其中国有及限额以上工业增长13.6%，综合效益指数116.4%，实现利润增长39.3%。第三产业发展良好，城乡市场比较活跃，社会消费品零售总额116亿元，增长10%。地方财政一般预算收入完成12.06亿元，为调整预算的104%，比上年实际完成增长21%。金融运行平稳，止年底，金融机构存贷款余额达247亿元和197亿元，分别比年初增长19.4%和7.2 %。

(二)结构调整步伐加快。三次产业结构调整为32.7∶39.8∶27.5，"二、一、三"产业格局更加稳定。农业结构调整取得新进展，优质小麦和优质玉米种植面积分别比上年增长3倍和1.3倍;板栗、花木、中药材、精细蔬菜等特色农产品产量都有较大增长;畜牧业持续发展，生猪、牛、羊饲养量继续保持全省领先地位。林业生产取得突破性进展，完成成片造林23.5万亩，建设和完善农田林网240万亩。大中型工业企业的支撑作用进一步增强，增加值占国有及限额以上工业的40%;药品、卷烟、化肥、水泥等主导产品的生产规模进一步扩大。第三产业继续发展，旅游业快速成长，接待游客168万人次，总收入1.4亿元，增长76.8 %;现代物流、社区服务等新兴服务业稳步发展，商贸、餐饮等传统服务业质量和档次有较大提高。

(三)基础设施建设得到加强。预计完成全社会固定资产投资76.5亿元，增长10.6%，其中基本建设投资完成23.68亿元。年初确定的50个重点项目完成投资30.18亿元，已建成投用18个，新开工及预备新开工项目进展顺利。公路建设完成投资12.3亿元，107和106国道改造等工程已建成通车，开龚路、南驻路、明临路等项目正在紧张施工。中心城区建设完成投资6.01亿元，是上年的2倍，练江路立交桥、污水处理厂等重点工程进展顺利，城市功能进一步完善，框架继续拉大。县城和小城镇建设取得新的突破。电力及邮电通讯业快速发展。农业基础设施建设步伐加快，小洪河、大洪河治理等重点项目进展顺利，全年新增有效灌溉面积32万亩，新增旱涝保收田20万亩。

(四)改革开放取得新的成就。农村税费改革取得阶段性成果，农民负担平均减轻37%。义务教育体制改革进展顺利，教育资源得到整合，布局趋于合理。国有企业改革继续深化，企业发展活力有所增强。县乡机构改革稳步推进，行政审批制度改革取得明显成效。对外开放进一步扩大，成功举办了2002年全国乡镇企业东西合作经贸洽谈会，落实签约项目567个，引进市外资金16.3亿元。全年完成出口总值2800万美元，增长44.7%，实现了食用小麦出口零的突破;实际利用外资增长2.5倍。

(五)社会事业全面进步。认真落实"科教兴市"战略，实施各类科技计划项目243项，组织推广优秀科技成果155项。基础教育得到巩固，"普九"人口覆盖面接近100%;高中教育得到加强，全市高中招生比上年净增1.4万人;职业教育、成人教育稳步发展;筹建本科院校步伐加快。计划生育管理和服务水平不断提高，人口自然增长率5.8‰，圆满完成了省下达的目标。文化艺术事业进一步繁荣。群众性体育活动健康发展。城乡医疗卫生服务体系建设得到加强。环保执法力度加大，环境质量得到改善。依法加强土地和矿产资源的保护和管理，促进了资源的合理开发和综合利用。广泛开展"双拥"和军民共建活动，增进了军政、军民团结。统计、人事、审计、民族宗教、广播电视、外事、侨务、气象、人防、史志等各方面都取得了新的成绩。

(六)人民生活继续改善。城镇居民人均可支配收入达到5303元，农民人均纯收入2079元，分别比上年增长12.4%和6.4%;城乡居民储蓄存款余额198亿元，比年初增加了29.3亿元。居民消费支出增加，结构得到改善。就业和社会保障工作力度加大，保证了企业离退休人员基本养老金、国有企业下岗职工基本生活费的发放，落实了城镇居民最低生活保障制度。完成了15万人口的脱贫任务， 47.4万受灾群众得到了及时救助。

(七)精神文明和民主法制建设进一步加强。广泛开展群众性精神文明创建活动，深入开展"双创"工作，城乡面貌和广大干部群众精神风貌发生了新的变化。坚持依法治市和依法行政，规范了政府行为。严格执行人大及其常委会通过的决定、决议，自觉接受人大法律监督和政协民主监督，认真办理人大代表议案和政协委员提案。积极推行村务公开、民主管理，圆满完成了第四届村民委员会换届选举任务。重视群众来信来访工作，切实解决群众反映的热点难点问题。健全安全生产管理体制，确保了安全生产形势的平稳。大力整顿和规范市场经济秩序，深入开展反腐纠风工作，坚持打防并举，加大了社会治安综合治理力度，维护了社会大局稳定。

各位代表，上述成绩的取得，是省委、省政府和市委正确领导的结果，更是全市人民共同努力的结果。在此，我代表市人民政府，向为我市经济建设和社会发展付出辛勤劳动的广大工人、农民、知识分子、干部、人民解放军指战员、武警官兵、政法干警和社会各界人士致以崇高的敬意!向关心、支持政府工作的各位人大代表、政协委员和民主党派、工商联、无党派人士表示衷心的感谢!

在肯定成绩的同时，我们也应清醒地看到，当前我市经济社会发展中还存在不少的矛盾和困难。主要是:国民经济整体竞争力不强，产业关联度低，结构性矛盾仍然比较突出;农业基础薄弱，标准化建设滞后，专业化程度不高;国有企业改革进展缓慢，部分企业尚未走出困境;县乡财政收支矛盾突出，保重点支出难度较大;社会保障水平不高，少数群众生活困难;社会投资尚未全面启动，个体、私营经济发展还不够充分;城市意识和城市管理水平有待提高;一些地方经济环境、治安状况和干部作风仍存在不少问题。对此，我们一定高度重视，在今后的工作中认真研究并加以解决。

折叠编辑本段2003年政府工作的总体要求和奋斗目标

综合分析当前和今后一个时期经济社会发展的形势，我们既面临严峻的挑战和困难，同时也拥有许多机遇和有利条件。党的十六大增强了全市广大干部群众奋起直追、加快发展的信心和决心;国家继续实施积极的财政政策和稳健的货币政策，有利于我们进一步扩大投资和消费需求;入世积极影响的显现和发达地区产业转移的加快，有利于我们进一步扩大对外开放;知识经济和新型工业化的兴起，给落后地区超越经济发展的阶段性限制，实现突破性发展提供了可能;目前我市正处于工业化全面加速的准备时期，潜力很大;近几年我们争取和实施了一大批工业、农业、交通等基础设施项目，又通过招商引资新上了一批东西合作项目，发展条件逐步改善，整体水平得到提高，具有充足的发展后劲。只要我们充分利用这些有利条件，抓住机遇，发挥优势，趋利避害，迎难而上，就一定能够实现我们的宏伟目标。

党的十六大提出了本世纪头20年全国国内生产总值翻两番的奋斗目标。围绕这一目标，省委提出到2020年人均国内生产总值力争接近或达到全国平均水平。我市作为中部欠发达的农业大市，与全省、全国的平均发展水平相比差距较大。要缩小差距，加快小康社会建设步伐,我们必须高于全国、全省的发展速度。只有这样，到2020年才能接近全省的平均发展水平。

要实现全市经济的快速健康发展，当前和今后一个时期，我们必须立足全市资源优势、区位优势和后发优势，解决好经济生活中存在的各种矛盾和问题，以工业化、城镇化和农业产业化为着力点，调整结构，深化改革，扩大开放，优化环境，力争经过一个时期的奋斗，把我市建设成为优质农产品生产和加工基地、东西部之间的产业转移基地、劳动力输出基地和旅游休闲基地，全面提高国民经济的整体竞争力。

今年是全面贯彻落实十六大精神的第一年，也是我市实施"十五"计划的关键年。根据中央、省委和市委有关经济工作的安排部署，今年政府工作的总体要求是:坚持以邓小平理论和"三个代表"重要思想为指导，认真贯彻党的十六大、中央、省委和市委经济工作会议精神，以全面建设小康社会统揽工作全局，继续坚持以发展为主题，以结构调整为主线，以改革开放和科技进步为动力，以提高人民生活水平为根本目的，加快两化进程(工业化、城镇化)，强化一个基础(农业)，实施三大战略(科教兴市、开放带动、可持续发展)，实现四个突破(东西合作、非公有制经济、畜牧业、旅游业)，保持国民经济快速健康发展和社会全面进步。

根据上述总体要求，兼顾"十五"计划和2020年奋斗目标，今年我市经济和社会发展的主要预期目标是:国内生产总值增长10%，其中第一产业增长5%，第二产业增长12.5%，第三产业增长12%;全社会固定资产投资增长11%;社会消费品零售总额增长10.5%;财政收入与经济发展保持同步增长;外贸出口增长12%;城镇居民人均可支配收入增长8%，农民人均纯收入增长5%;人口自然增长率控制在7‰以内;城镇登记失业率控制在4.5%以内。

实现上述目标，必须坚持把发展作为第一要务，解放思想，实事求是，与时俱进，切实做到发展要有新思路，改革要有新突破，开放要有新局面，各项工作要有新举措。必须坚持速度和结构、质量、效益相统一，既要保持较高的增长速度，又要特别注重提高经济增长的质量和效益;必须正确处理改革、发展、稳定的关系，坚持把改革的力度、发展的速度和社会可承受的程度统一起来，以改革促发展，以发展保稳定;必须优化资源配置，采取有效措施，积极解决资源闲置和浪费问题，切实把我们有限的资源配置好;必须立足我市现有条件，通过上大项目，培育加工龙头企业，拉长产业链条，增强产业关联度，促进基础产业快速发展;必须完善区域发展规划，相对集中，搞好区域分工，努力培育区域经济规模优势，提高工农业生产的专业化程度;必须逐步建立健全农产品质量安全标准体系、检测体系、认证体系以及研究推广体系，不断提高农业标准化水平。坚持上述原则，必须做到既尽力而为，又量力而行，积极稳妥地推进各项事业的发展。

折叠编辑本段2003年政府工作的主要任务

(一)调整结构，提高经济运行的质量和效益

1.调整优化工业结构，走新型工业化道路

坚持运用高新技术和先进适用技术改造传统产业，有选择地发展高新技术产业，鼓励企业应用信息技术，提高装备水平和信息化程度，促进工业升级，走新型工业化道路，加快工业化进程。立足我市资源优势和现有工业基础，积极发展适应多元化市场需求的产品，大力培植名牌产品，努力培育食品、制药、建材、卷烟、化工、机械电子、轻纺等主导产业。加大企业技术改造力度，以扩大优质产品生产规模为重点，抓好技改项目的申报和实施工作，确保项目进度和质量，推进企业升级和产品更新换代。

推进企业组织结构调整，突出抓好天方药业、中原气化、驻马店卷烟、骏马化工、银马纺织、白云纸业、金雀电气、豫南水泥、华骏车辆、十三香集团、顶志集团、上蔡荣光、遂平华强、汝南双星等骨干企业，打造我市优势企业群体，增强工业经济抗御风险能力。充分利用人力资源，大力发展劳动密集型产业，逐步形成劳动密集型产业与资金技术密集型产业优势互补、协调发展的产业格局。继续依法关闭产品质量低劣、浪费资源、污染严重的企业，逐步淘汰落后生产能力。

2.调整城乡结构，加快城镇化进程

坚持以市区为龙头，以县城为骨架，以小城镇为基础"三级联动"、"三头并举"的方针，加速推进城镇化。强化城镇规划控制。充分发挥城市规划在城市建设中的龙头作用，严格执行中心城区总体规划，认真编制详细规划、专业规划和街景规划，确保局部规划与整体规划的协调统一。积极引导支持各县城和小城镇根据区位优势、自然条件，突出特色，因地制宜，合理布局，科学规划。加强规划管理，规范审批程序，坚决维护规划的严肃性、权威性。

加快城镇基础设施建设。突出抓好中心城区六路一河一桥两场等一批城市基础设施建设项目，积极创造条件，做好行政区北迁前期准备等工作，加快旧城改造和新城区开发建设步伐，继续拉大中心城区框架。对城镇水、电、路、园、区等设施进行提升改造，进一步完善城镇功能。突出地方特色、文化特色和艺术特色，搞好城区景观建设。抓好城市绿化工作，提高城镇绿化率。抓好西平城镇化试点县建设，加快推进其县改市步伐。

加强城镇管理。以"双创"活动为载体，继续在全市范围内开展城镇环境整治和综合治理，促进城市建成区特别是中心城区交通秩序、环境卫生等进一步好转。强化城市意识，提高市民的文明程度，增强市民参与城镇管理的积极性。认真落实"三定"、"三包"责任制，形成上下协调运作，齐抓共管的城镇管理格局。积极推广平舆城市建设和管理经验，全面提高城镇管理水平。

增强经营城市意识。通过出让、转让、拍卖、特许经营等多种形式，盘活城市基础设施存量和增量资产。认真落实城市建设用地"五统一"制度，政府垄断土地一级市场，规范二级市场，加大土地违法违纪查处力度，确保土地收益，走"以城建城、以城养城、滚动发展"的路子。

培育城镇支柱产业。立足自身优势，注重发展城镇支柱产业和特色经济，形成产业支撑，大量吸纳农村富余劳动力，提高城镇的聚集和辐射能力，加快城乡一体化进程。

3.调整农业结构，全面发展农村经济

全面建设小康社会的重点和难点在农村，实现工业化的重点和难点也在农村，没有农民的小康就没有全市人民的小康。作为农业大市，我们一定要立足增加农民收入，始终把农业、农村、农民问题作为重中之重，放在更加突出的位置，不断强化农业的基础地位，努力开创农村、农业工作新局面。坚持用发展工业的理念发展农业，围绕农产品生产和加工基地建设，把农业结构调整的重点由作物结构的适应性调整转移到品种结构、品质结构、区域布局结构和农村经济结构的战略性调整上来。稳定粮食生产能力，突出发展生态农业、特色农业、品牌农业和订单农业。继续扩大优质小麦、优质玉米规模化种植，大力发展蔬菜、食用菌、花木、中药材、烟叶等高效经济作物，促进大宗农产品的优质化和特色农产品的多样化、规模化。因地制宜发展林果业，建设优质种苗生产基地，加大植树造林力度，提高林业在农业中的比重。进一步优化畜牧业结构，在保持生猪生产优势的同时，突出抓好牛羊生产，大力发展奶业生产;继续推广优良畜禽品种，合理布局养殖业发展区域，加快养殖业基地和规模养殖企业发展步伐，促进畜牧业优化升级。着力培育和壮大龙头企业，大力发展农畜产品精深加工业、冷藏保鲜业以及农村服务业。搞好区域布局和分工，提高农业组织化程度和专业化水平，积极推进农业产业化。按照"以点带面，强力推进"的原则，加强农业标准化建设和农产品质量安全管理工作，促进标准农业、创汇农业的发展，增强农产品在国内外市场的竞争力。推进乡镇企业改组改造，优化产品结构，加强企业管理，引导乡镇企业向小城镇集中，加快农村工业化进程。

4.大力发展服务业，提高第三产业比重

坚持以旅游业、房地产业和新型服务业为重点，全面发展第三产业，提高第三产业在国民经济中的比重。加快旅游业发展。围绕休闲旅游基地建设，继续以"铸精品、创品牌、树形象"为目标，以"双创"活动为载体，突出抓好"一带四区"景点开发和配套基础设施建设，搞好宣传推介工作，巩固老市场，开发新市场，打造"天之中"生态文化旅游品牌，保持旅游客源继续强劲增长。创新旅游业投融资机制，鼓励和支持多种经济成份参与旅游开发和经营，促进旅游业快速发展。

大力发展服务业。稳定发展交通运输、邮电通讯、商业、餐饮等传统服务业，积极发展信息、咨询、会计、审计、法律等中介服务和保险服务业，引导文化娱乐、教育培训、体育健身、卫生保健等产业发展。积极争取省服务业发展引导资金，加强社区服务设施和网点建设，推动社区服务业快速发展。大力培育特色市场，尤其是各类专业批发市场，增强辐射、带动功能。积极探索现代物流方式，推行连锁经营、特许经营、物流配送、电子商务等流通形式，努力开拓市场。扩大服务业对外开放，吸引国内外知名企业来我市开展业务，以提高服务业整体水平。

积极发展房地产业。鼓励支持商品房开发，扶持发展经济适用住房。扩大住房信贷规模，为居民购房提供信贷支持。规范房产交易市场，适时开放二级市场。积极发展家居装修、物业管理等房产伴生行业，提高住房建设水平。

5.大力发展非公有制经济，培育新的经济增长点

毫不动摇地发展非公有制经济，放宽非公有制经济市场准入领域，打破行业垄断，凡国家没有明令禁止的，都允许个体私营资本进入。全面落实促进非公有制经济发展的各项政策，消除影响非公有制发展的各种障碍，使非公有制经济在投融资、税收、土地使用和对外贸易等方面实现公平竞争，在政策上一视同仁，继续提高非公有制经济在国民经济中的比重。

正确引导非公有制经济发展，鼓励个体私营经济充分利用本地资源，向一、二产业进军，向基础建设领域开拓，向生产加工型、外向型、科技型发展;引导非公有制企业通过各种形式参与国有企业改组、改制，与公有制经济互相融合、渗透资本、优势互补、发展规模经济。支持那些发展前景广阔的非公有制企业走集团化道路，向规模经济迈进，快速发展壮大。

加强对非公有制企业、个体工商户的监督和管理，教育引导经营者树立诚信守法、公平竞争、勤劳致富、奉献社会的新形象。高度重视非公有制经济发展环境问题，依法保护非公有制经济的合法权益，努力为非公有制经济发展创造安全、公平、宽松的环境。

(二)扩大需求，促进经济持续快速增长

加大投入，促进消费，是拉动经济快速增长最直接、最有效的途径，也是我们缩小差距，加快发展的重要手段。

1.加强基础设施建设，扩大投资需求

以项目建设带动投资增长，以扩大投资培育新的经济增长点，扩充经济总量，是我市实现经济快速发展的必然选择。

切实做好项目储备和申报工作。各级政府要高度重视项目工作，把更多的力量投入到项目建设中去。抓住国家继续实施积极财政政策和国债资金向农村、向结构调整、向中西部、向生态环境建设倾斜的机遇，加大项目工作力度，抓紧论证、储备和上报一批对全市经济社会发展具有拉动作用的大项目、好项目，努力争取国家和省更多的资金支持。

抓好重点项目建设。突出抓好55个投资3000万元以上的重大项目。工业项目建设方面:确保驻马店卷烟厂"九五"技改、华骏工业园和华强二期扩建等工程按期竣工;确保白云纸业达产;加快骏马化工复合肥和天方药业技改等项目建设;争取吴桂桥煤矿、30万吨甲醇等项目前期工作取得实质性进展;保证古城电厂2×30万千瓦以大代小技改、天中面粉实业有限公司小麦深加工、豫南天瑞水泥有限公司日产5000吨熟料生产线、天方药业新药生产线四个项目，完成年度工作计划。城市基础设施建设方面:加快市热电厂集中供热工程、西气东输豫南支线工程建设步伐;突出抓好市区"六路、一河、一桥、两场"项目建设，分步实施冷水河的拓宽整治，确保练江路立交桥建成通车。农林水方面:抓好以中小河流的拦蓄、平原打井配套以及四大灌区的修复改造为主要内容的旱保田建设;加快优质小麦基地、大小洪河治理等项目建设，确保完成年度工程进度;加大植树造林力度，抓好绿色通道工程和淮河防护林工程建设，完成退耕还林8万亩和荒山荒地造林15.5万亩任务。公路建设方面:保障驻信高速公路建成通车;抓好2002年13个干线公路项目的实施，确保按时建成通车;开工建设驻信高速公路驻马店南和确山两条连接线，并保证与驻信高速同步建成;完成北二环、北三环西段、雪松路西段、西三环北段的配套工程和练江路的拓宽改造工程;推进县乡公路升级项目建设;完成两个县的村村通工程。电力方面:全面完成城乡电网改造二期工程。

加强项目协调和监管。完善项目法人责任制、工程质量领导责任制和招投标制，加强监督，严格程序，规范操作，坚决制止规避招投标和违法分包等行为，确保各类投资项目的进度和质量。

2.增加城乡居民收入，扩大消费需求

拓宽城镇就业渠道。就业是民生之本。坚持劳动者自主择业、市场调节就业、政府促进就业的方针，以增加就业岗位为目标，创造更多的就业机会。大力发展中小企业和劳动密集型产业，注重发展就业容量大的物业管理、托幼养老、家政等社区服务业。支持国有企业通过主辅分离、辅业转制等多种方式安置富余人员。引导下岗失业人员转变就业观念，鼓励和提倡自谋职业、自主创业。全面落实下岗失业人员再就业的各项优惠政策，帮助下岗失业人员再就业。加强就业培训和就业服务，积极开展对城镇就业困难群体的就业援助。完善劳动力市场体系，规范和发展各类职业技术教育。

加强社会保障工作。切实落实社会保障政策，确保国有企业下岗职工基本生活费和企业离退休人员养老金按时足额发放。规范城市"低保"，合理确定"低保"对象和"低保"标准，切实做到应保尽保。做好"三条保障线"的衔接工作，积极稳妥地推进下岗职工基本生活保障与失业保险并轨。继续扩大社会保险覆盖面，强化社会保险费的征缴和支出管理。完善对低收入者的救助制度，采取多种帮助措施，切实解决城镇困难家庭的实际问题。

增强农村居民消费能力。大力发展农村二、三产业，引导农民向非农产业转移。围绕建立劳动力转移基地，加强劳动力培训，逐步建立劳动力转移服务网络，组织好农村劳动力输出。妥善解决费税改革后出现的新情况、新问题，巩固税费改革成果，防止农民负担反弹。切实做好扶贫救灾工作，努力完成15万贫困人口的脱贫任务，确保所有困难群众有饭吃、有衣穿、有房住，不让一个学生因贫困而失学。

3.加强财政金融工作，为扩大需求创造有利环境

大力培植财源。加强二、三产业财源建设，扩大税基，确保财政收入稳定增长。坚持依法理财，调整财政支出结构，严格控制、压缩会议费、招待费等一般性支出，优先保证工资发放、社会保障资金和机关工作正常运转等重点支出。牢固树立勤俭节约、艰苦奋斗的思想，反对奢侈浪费，把有限的资金用到群众生活和重点建设等急需的地方。加强财税监管，落实县乡财政管理体制，完善部门预算、国库集中收付和政府采购制度。严格执行"收支两条线"规定，规范财政收支行为。坚持依法治税，加大征管力度，严厉打击偷逃税款行为，做到应收尽收。

认真贯彻执行金融政策，充分发挥金融对扩大需求的支持作用。强化信用意识，促进企业与银行建立互信互助的银企关系。引导金融机构不断扩大信贷规模，优化贷款结构，增加对技术改造、结构调整、重点建设和科技企业的资金支持。支持金融机构整顿金融秩序，防范化解金融风险，防止金融风险转化为财政风险。增强保险意识，促进保险业有序快速发展。

(三)深化改革，增强经济发展活力

继续按照"积极稳妥、分步实施、一厂一策、强力推进"的原则，深化国有企业改革，推进企业的体制、技术和管理创新。突出抓好市直重点企业的产权制度改革，通过健全法人治理结构、规范上市、互相参股、对外出售等途径加快现代企业制度建设步伐，实现股权多元化。认真贯彻落实国家、省鼓励和促进中小企业发展的各项政策，继续放开搞活国有中小企业，推进中小企业向"专、精、特、新"方向发展。县及县以下所属企业原则上不再保留纯国有形式。完善市场退出机制，对长期亏损、资不抵债、扭亏无望的企业坚决依法实施破产。

坚持党在农村的基本政策。依法稳定农村土地承包关系，完善土地征用制度，严厉查处各种侵害农民土地承包权益的违法行为。按照依法、自愿、有偿的原则进行土地承包经营权流转，逐步发展规模经营。全面落实农村税费改革政策，搞好配套改革，巩固和扩大税费改革成果，确保农民负担不反弹。继续深化粮食流通体制改革，争取和用好补贴资金。深化水利和林业产权制度改革，提高水利和林业效益。

深化行政审批制度改革。坚决取消妨碍市场开放和公平竞争的行政审批事项，依法规范行政审批行为，加强对已经取消的审批项目的后续监管工作，防止反弹或者变相增加新的审批项目。积极推行政务公开制、服务承诺制和限时办结制，提高服务质量和效率。

逐步建立和形成多元化的投融资机制。充分发挥市场机制的作用，进一步打破行业和部门垄断，鼓励各种投资主体平等参与、公平竞争。注重利用产权、经营权和价格机制融资，以存量换增量，允许各类投资主体参股、控股。积极利用资本市场融资，着力培育上市后备资源;采取切实有效措施，吸引和鼓励民间投资。

(四)加快开放步伐，提高经济外向度

继续推进东西合作，巩固和扩大东西合作成果。突出抓好洽谈会签约项目的履约和资金到位工作。加强在建项目协调、管理，加快项目建设进度。坚持市场运作，创新办会路子，搞好联络衔接，积极争取举办2003年全国东西合作会，进一步展示形象，扩大战果。强化招商引资工作，加强同明星企业、大专院校、科研院所的联合与合作，围绕产业转移基地建设，积极吸引和承接东部转移的产业和行业，加快我市优势资源的开发利用步伐。鼓励和支持有条件的企业到西部承包工程、投资办厂或建立销售网点，拓展企业发展空间。切实保护外来企业合法权益，引导合作企业不断追加投资，壮大企业规模，提高市场竞争力。

努力扩大外贸出口，加强外贸人员培训，认真研究分析国际市场形势，优化出口商品结构。继续支持天方药业、康茂、荣光鞋业等企业的产品出口，扩大非农产品出口规模;加快优质小麦、芝麻、花生、生猪、香菇等农畜产品出口基地建设，扩大优质农产品出口。实施市场多元化战略，鼓励各类所有制企业开展出口业务，获得出口经营权。积极开拓国际劳务市场，加强对劳务输出的组织和管理，开展不同形式的对外劳务合作。积极开展对外商务活动，扩大引资领域和利用外资规模。

努力为扩大开放创造良好的环境。加强社会信用体系建设，健全信用制度，依法惩处破坏信用秩序的行为。继续开展打假打诈联合行动，严厉打击制售假冒伪劣等违法犯罪活动。深入开展各类专项整治工作，打破地方保护和行业垄断，促进市场的统一、开放和竞争有序。继续治理经济环境，进一步清理收费项目，规范收费行为。坚决制止对企业进行乱摊派、乱收费、乱罚款、乱检查，切实保护企业和投资者的合法权益。对顶风违纪、吃拿卡要的单位和个人，要发现一起，从重从快从严查处一起，决不姑息迁就。

(五)实施"科教兴市"和可持续发展战略，全面发展各项社会事业

加快创新步伐，推动科技进步。发挥"第一生产力"的重要作用，注重依靠科技进步，提高经济增长质量和效益，用高新技术改造提升传统产业。认真组织实施农业科技攻关，积极推进农业科技革命。大力发展高新技术产业，加大科技对外合作力度，积极争取省、国家高新技术产业化项目。注重高新技术的引进、消化和吸收，加速科技成果向现实生产力的转化，为新型工业化提供技术支撑。加大科技投入和对高新技术项目的支持力度，鼓励技术创新，建立和完善企业自主创新机制，使企业成为技术进步和创新的主体。建立健全科技服务体系，发展技术市场和科技中介组织，促进产学研结合。加强知识产权保护和人才的培养与引进，尊重劳动、尊重知识、尊重人才、尊重创造，营造有利于人才汇集、人才创业、人才发展的良好环境。办好高新技术产业开发区，理顺体制,落实政策,增强其对资金、人才、产业的聚集能力。

大力发展教育事业。全面贯彻党的教育方针，坚持把教育摆在优先发展的战略地位。加快教育结构和布局调整，深化农村义务教育管理体制改革。切实加强基础教育，增加教育投入，确保教师工资发放。巩固提高"两基"成果，提高义务教育办学水平;加快发展高中阶段教育，扩大招生规模。积极发展职业教育，优化配置教育资源，促进普通高中和中等职业教育协调发展。开展教育创新，推进素质教育。加强高素质教师队伍建设，全面提高教学质量。鼓励、支持社会力量办学，大力发展民办教育。积极发展高等教育，加快本科院校申办工作。

实施可持续发展战略。加强人口控制、资源管理和环境保护，促进经济社会协调健康发展。全面落实人口与计划生育目标管理责任制，确保实现全市人口控制目标，稳定低生育水平。合理开发、节约使用和依法保护好水、土地、矿产、森林、野生动物等资源。加强对工业污染、城市生活污染的治理，积极协调解决小洪河水污染治理问题。认真落实"三同时"制度，严格控制新污染源的产生。改善人居和生态环境，提高城乡居民生活环境质量。

积极推进文化、卫生、体育等社会事业发展。支持公益文化事业发展，深化文化体制改革，加快文化场馆和广播电视基础设施建设，开工建设市图书馆，扩建市群众艺术馆，逐步改变文化设施落后状况。强化文化市场管理，繁荣文学艺术、新闻出版、广播影视，实施"广电网络建设年"工程，开展各类群众文化活动，满足人民群众日益增长的文化生活需求。保护利用文物和文化遗产，继承和发扬民族传统文化。推进城镇医疗体制改革，加快发展城市社区卫生服务;加强农村卫生工作，建立和完善农村医疗卫生体系，改善城乡卫生条件，提高医疗服务水平。加大卫生监督执法力度，继续做好传染病的控制和预防，抓好卫生县城的创建活动。加快体育设施建设，发展竞技体育，开展全民健身活动，提高全民健康水平。加强国防教育，落实优抚政策和转业退伍安置政策，密切军政军民关系，切实做好"双拥"工作，争创全国"双拥"模范城。重视统计工作，提高对经济运行的分析、监测、预测水平。做好救灾、救济工作，妥善安排好灾区群众生产生活。认真落实各项保障政策，切实做好弱势群体的救助工作。依法保护妇女儿童、残疾人和未成年人的合法权益。继续做好民族宗教、外事侨务、人事、审计、人防、气象、史志、红十字会等工作。

(六)加强社会主义精神文明和民主法制建设，努力保持社会稳定

切实加强思想道德建设。认真贯彻《公民道德建设实施纲要》，加强社会公德、职业道德、家庭美德教育，倡导尊老敬贤、诚实守信、见义勇为的良好风尚。认真组织科技、文化、卫生"三下乡"活动，普及科学思想和科学知识。以"双创"为载体，广泛开展群众性精神文明创建活动，集中抓好窗口行业和执法部门的精神文明建设，营造积极向上、文明健康的社会氛围。

积极推进民主法制建设。自觉接受人大及其常委会的监督，认真执行人大及其常委会通过的各项决定、决议;自觉接受政协民主监督，加强与民主党派、工商联和无党派爱国人士的沟通。认真办理人大代表议案、建议和政协委员提案。积极支持工会、共青团和妇联等人民团体工作。加强基层民主法制建设，健全民主管理制度。搞好普法教育，增强全市干部的法制观念。加强政府法制建设，坚持依法行政，推进依法治市进程。

努力保持社会稳定。高度重视安全生产和安全监察，强化安全生产责任制，严格执行重特大事故行政责任追究的规定，坚决遏制重特大事故的发生。依法加强民间组织管理。高度重视人民群众来信来访工作，妥善处理各种群体性事件。加强社会治安综合治理，深入开展严打整治斗争，坚决查禁黄、赌、毒等社会丑恶现象。特别是对于那些杀人、抢劫、盗窃以及带有黑社会性质的团伙犯罪和地痞流氓恶势力，要重拳出击，除恶务尽，切实增强人民群众的安全感，真正履行保一方平安的神圣职责。

政府是人民的政府。人民政府的根本职责就是全心全意为人民服务。在新世纪、新阶段，面对时代赋予的庄严使命，肩负全市800多万人民的重托，要全面完成今年的各项目标任务，就必须切实加强政府自身建设，不断提高政府的工作效率和工作水平，努力建设勤政、廉洁、务实、高效、诚信的政府，建设法制、有限、服务、阳光、责任的政府。全市各级政府的工作人员都要加强学习，更新观念，提高自身政治素质和业务素质。要牢固树立宗旨意识、公仆意识和执政为民意识，深怀爱民之心，常谋富民之策，多办利民之事。要务必继续保持谦虚谨慎、不骄不躁的作风，务必继续保持艰苦奋斗的作风，深入基层，深入群众，体察民情，倾听民声，切实为群众排忧解难，实实在在地为群众办事，为群众办实实在在的事。要强化工作责任，狠抓工作落实，科学合理地搞好目标任务分解，加强督促检查和跟踪问效，确保政令畅通和责任落实。要树立正确的政绩观，坚决反对各种脱离实际的"形象工程"、"政绩工程"，坚决反对形式主义、官僚主义。要坚持依法行政，从严治政，降低行政成本，规范政府行为，不断提高行政效率和执政水平。要坚持标本兼治、综合治理，加强政府廉政建设和反腐败斗争，努力从源头上预防和解决腐败问题。要努力营造干事创业的浓厚氛围，扎扎实实地推进各项工作，努力使工作作风有较大转变，干群关系有较大改善，行政水平有明显提高，集中全市人民的智慧，凝聚全市人民的力量，圆满完成今年的各项任务。

各位代表，党的十六大为我们描绘出了小康社会的美好蓝图。在驻马店这个基础薄弱、百业待兴的农业大市,把蓝图变为现实,是一个艰苦卓绝的奋斗过程,也是一项激动人心的伟大事业，更是我们责无旁贷的历史使命。让我们在省委、省政府和市委的领导下,在市人大、政协的监督支持下,高举邓小平理论伟大旗帜，按照"三个代表"重要思想的要求，进一步解放思想,振奋精神,同心同德，务实苦干，为全面振兴驻马店，开创我市社会主义现代化建设新局面而努力奋斗!
